# Enhancement of the Follicular Lymphoma International Prognostic Index (FLIPI) with lymphopenia (FLIPI-L): a predictor for overall survival and histologic transformation

**DOI:** 10.1038/s41408-019-0269-6

**Published:** 2020-01-02

**Authors:** George Yang, Matthew Mills, Youngchul Kim, Nicholas B. Figura, Catherine Doyle, Daniel Oliver, G. Daniel Grass, Timothy Robinson, Julio Chavez, Sungjune Kim

**Affiliations:** 1 Department of Radiation Oncology, H. Lee Moffitt Cancer Center and Research Institute, Tampa, FL 33612 USA; 2 University of South Florida, Morsani College of Medicine, Tampa, FL 33612 USA; 3 Department of Bioinformatics, H. Lee Moffitt Cancer Center and Research Institute, Tampa, FL 33612 USA; 4 Department of Malignant Hematology, H. Lee Moffitt Cancer Center and Research Institute, Tampa, FL 33612 USA; 5 Department of Immunology, H. Lee Moffitt Cancer Center and Research Institute, Tampa, FL 33612 USA

**Keywords:** B-cell lymphoma, B-cell lymphoma

Dear Editor,

Follicular lymphoma (FL) accounts for one-third of adult non-Hodgkin’s lymphomas (NHLs) in the Western hemisphere. Treatment, including observation, chemoimmunotherapy, surgery, or radiation, is guided by several prognostic indices^1^.

The original Follicular Lymphoma International Prognostic Index (FLIPI) was created from five clinicobiological features and defines low (0–1), intermediate (2), and high (3–5) risk groups with 5-year overall survival (OS) of 91%, 78%, and 53%, respectively^2^. Other prognostic indices have been modified FLIPI, adding cardiovascular disease^3^ or β2-microglobulin and bone marrow involvement in PRIMA-PI^4^. Finally, molecular models (m7-FLIPI) use mutational status based on next-generation sequencing of seven genes^5^.

Absolute lymphopenia has prognostic consequence in several hematologic malignancies and solid tumors^6,7^. The impact of lymphopenia in diffuse large B cell lymphoma (DLBCL) suggests enhanced risk stratification in FL, given the intertwined carcinogenesis and potential for transformation. Prior studies have demonstrated that lymphopenia was associated with worsened OS in FL^8^. Therefore, we hypothesized that lymphopenia may be integrated with existing FLIPI to better stratify long-term survival outcomes and predict for transformation.

After institutional review board approval/informed consent waiver, patients at our institution diagnosed with FL between January 1999 and April 2016 were retrospectively reviewed. Clinicopathologic characteristics and absolute lymphocyte count was abstracted from the medical record. Absolute lymphopenia was defined as <1.0 × 10^9^/L. Transformation to high-grade DLBCL, the presence of double hit confirmed with *myc* fluorescent in situ hybridization (FISH), and the presence of double expressor phenotype (>40% *myc* overexpression on immunohistochemistry (IHC)) were documented.

OS was the primary endpoint, defined from diagnosis until death or last follow-up. Survival analysis was performed using Kaplan–Meier log-rank method. Continuous and categorical variables were compared using the Mann–Whitney *U* and Chi-square analysis. All tests were two-sided; *p* < 0.05 was significant (SPSS v24 IBM, Armonk, NY).

Cox proportional hazard analysis was used to build a proportional-hazard model incorporating FLIPI and absolute lymphopenia. The prognostic index was derived from the addition of absolute lymphopenia to FLIPI.

The FLIPI with lymphopenia (FLIPI-L) model (OS) used standard *k*-fold cross-validation and split the initial cohort into four distinct groups. To calculate the predictive strength of the new FLIPI-L models, patients were randomly partitioned into four subsets of approximately equal size. This was subsequently fit to each three-quarter subset of the data and tested on the final quarter of the data, calculating mean area under the curve (AUC) for ability to predict OS/transformation.

Seven hundred and thirty-six patients were identified who had all available data with a median follow-up of 72 months (range 2–211, interquartile range (IQR) 40–120 months). Table 1 contains patient/treatment characteristics.

**Table 1:** Patient characteristics.

Patient characteristic	Not lymphopenic		Lymphopenic		*p* value
	*N*	(%)	*N*	%	
Age					
≤60 years	291	54.8%	132	54.8%	0.994
>60 years	240	45.2%	109	45.2%	
LDH elevated					
No	435	86.1%	158	68.4%	<0.01
Yes	70	13.9%	73	31.6%	
Hemoglobin					
≥12 g/dL	437	83.6%	143	60.1%	<0.01
<12 g/dL	86	16.4%	95	39.9%	
Stage					
I/II	169	31.9%	50	20.8%	<0.01
III/IV	361	68.1%	190	79.2%	
Number of nodal sites					
≤4	376	70.8%	132	55.0%	<0.01
>4	155	29.2%	108	45.0%	
FLIPI score					
0	68	13.5%	8	3.5%	<0.01
1	148	29.3%	43	18.6%	
2	184	36.4%	73	31.6%	
3	68	13.5%	67	29.0%	
4	32	6.3%	31	13.4%	
5	5	1.0%	9	3.9%	
Grade					
1	261	51.6%	100	43.7%	0.10
2	169	33.4%	84	36.7%	
3	76	15.0%	45	19.7%	
Initial therapy					
Rituximab alone	55	10.4%	9	3.7%	<0.01
Rituximab+chemotherapy	221	41.6%	150	62.2%	
Radiotherapy	47	8.9%	14	5.8%	
Chemotherapy alone	44	8.3%	35	14.5%	
Surgery alone	11	2.1%	4	1.7%	
Observation	135	25.4%	23	9.5%	
Other	4	0.8%	0	0.0%	
Unknown	14	2.6%	6	2.5%	

The 5- and 10-year OS for the patient cohort was 81.3% and 67.3%, respectively. Five-year OS for low, intermediate, and high-FLIPI was 91%, 82.7%, and 66% respectively, and 10-year OS was 80.4%, 66%, and 45.8% (log-rank *p* < 0.01; Fig. 1c).

**Fig. 1:**
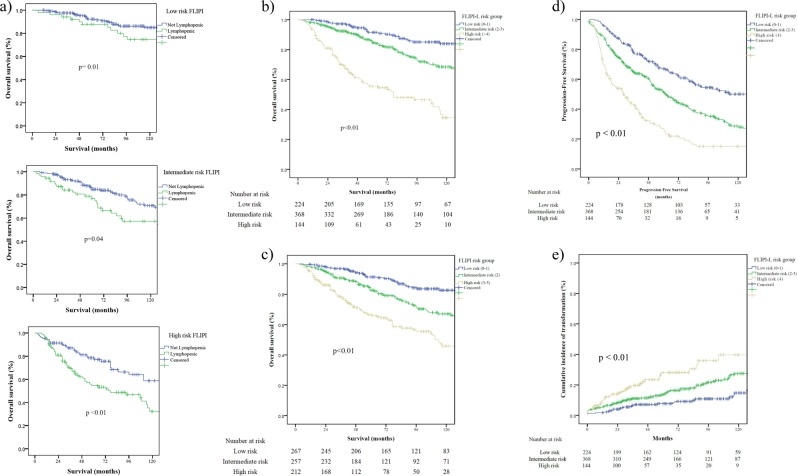
Overall survival and progression-free survival of FLIPI with lymphopenia and FLIPI-L risk stratification. **a** FLIPI risk groups segregated by lymphopenia score. **b** FLIPI-L Kaplan–Meier overall survival comparison by risk group. **c** FLIPI Kaplan–Meier overall survival comparison by risk group. **d** FLIPI-L progression-free survival by risk group. **e** FLIPI-L risk group cumulative incidence of transformation.

Patients with lymphopenia had worse 5- and 10-year OS, 69.5% vs 86.7% and 51.0 vs 74.0%, compared to those without lymphopenia, respectively, as well as inferior 5- and 10-year survival by FLIPI risk grouping (*p* < 0.01; Fig. 1a).

Patients with FLIPI/risk groups of 0–1 (low), 2 (intermediate), and 3–5 (high) had 5-year OS of 94.2%, 88.3%, and 71.6% and 10-year OS of 82.7%, 67%, and 45.8%, respectively (*p* < 0.01). Patients with FLIPI-L 0–1 (low risk), 2–3 (intermediate risk), and 4–6 (high risk) had 5-year OS of 94.5%, 89%, and 61% and 10-year OS of 83.9%, 68.5%, and 34.5%, respectively (*p* < 0.01; Fig. 1). Patients with FLIPI-L low, intermediate, and high had 2-year progression-free survival (PFS) of 88%, 75%, and 53.8% respectively (*p* < 0.01; Fig. 1d).

On univariate and multivariate analysis (UVA and MVA, respectively) lymphopenia was an independent predictor for OS (UVA hazard ratio (HR) 2.2 95% confidence interval (CI) 1.7–2.9, *p* < 0.01; MVA HR 1.74 95% CI 1.3–2.3, *p* < 0.01; Supplementary Table 1).

A simplified two-variable model score implementing calculated FLIPI and absolute lymphopenia was identified. The enhanced FLIPI was constructed and termed FLIPI-L, assigning one point for the standard FLIPI components and included one point for absolute lymphopenia (Distribution in Supplementary Table 1). FLIPI-L discriminated patients with different OS (log-rank *p* < 0.01). On univariate Cox regression analysis, increasing FLIPI-L was associated with increasing HR at each level (*p* < 0.02 for all) with minimum HR of 3.4 for FLIPI-L of 1 and maximum HR of 30.9 for FLIPI-L of 6 (Supplementary Table 2).

When evaluating patients who received rituximab or radiation alone or rituximab+chemotherapy, FLIPI-L remained prognostic for OS between low-, intermediate-, and high-risk groups (*p* < 0.04). For patients who were observed, FLIPI-L distinguished between low/intermediate- and high-risk groups (*p* < 0.01).

For patients with progression of disease within 24 months, FLIPI-L was more predictive of PFS, with estimated median event-free survival of 14.75, 12.55, and 10.0 months for FLIPI-L low, intermediate, and high, respectively (*p* = 0.05), while the FLIPI did not achieve significance (*p* = 0.11).

One-hundred and thirty-five patients (18%) experienced transformation to a high-grade lymphoma with a median time to transformation of 32 months (IQR 13.7–75 months). Patients with transformed disease had lower hemoglobin, elevated lactate dehydrogenase, and more stage III/IV disease, reflecting FLIPI >2 (all *p* < 0.001). In addition, transformed patients were more likely to be lymphopenic (48.9% vs 27.5%, *p* < 0.001). Patients were at higher risk for transformation based on their FLIPI-L risk categorization (*p* < 0.01; Fig. 1e).

Thirty-four (25.2%) patients with transformation had FISH analyses for *bcl2/myc* or *bcl6/myc* translocations performed—15 of the 34 (44%) had FISH-confirmed double-hit or triple-hit mutations. Three patients with transformation had *myc* overexpression (>40% on IHC).

Lymphopenia was an independent predictor of transformation on both univariate (odds ratio (OR) 2.53 95% CI 1.73–3.70, *p* < 0.01) and multivariate logistic-regression (OR 2.1 95% CI 1.4–3.1, *p* < 0.01; Supplementary Table 3). Neither the original FLIPI nor grade was an independent predictor of transformation. When lymphopenia was implemented, increasing FLIPI-L was an independent predictor of transformation when analyzed both as a continuous variable (OR 1.34, 95% CI 1.17–1.54, *p* < 0.01) and stepwise for FLIPI-L 3–5 (Supplementary Table 4).

The AUC for FLIPI-L (60 months) was 0.69 (95% CI 0.603–0.731) compared to an AUC 0.633 (95% CI 0.583–0.713) for the original FLIPI. The AUC for FLIPI-L (120 months) was 0.689 (95% CI 0.613–0.740) and AUC for FLIPI was 0.675 (95% CI 0.601–0.725). The FLIPI-L model for transformation was AUC 0.614 for FLIPI-L and 0.577 for FLIPI with overlapping 95% CI (Supplementary Fig. 1).

Cancer immunotherapy has brought about paradigm shift in cancer care. Lymphomas as an immune-hot tumor type are the forefront of innovation with immune checkpoint inhibitors^9^ and cell-based therapies such as chimeric-antigen receptor T cell therapy^10^. Because cancer immunotherapy critically depends on a functional immune system, there is renewed interest in the lymphocyte compartment. Our analysis of a lymphopenia cut-off as an addition to the original FLIPI is simple, yet improves risk stratification to differentiate between prognostic groups and, importantly, to predict transformation.

In this report, we demonstrate lymphopenia as a crucial prognostic biomarker. Lymphopenic patients manifest a significantly worse prognosis at every risk stratification by FLIPI. Strikingly, the survival for low-risk FLIPI with lymphopenia overlaps with that of intermediate-risk FLIPI. Likewise, the survival for low-risk FLIPI with lymphopenia overlaps with that of intermediate-risk FLIPI. These results suggest that FLIPI alone is insufficient to accurately estimate prognosis of patients with lymphopenia and thus provide a compelling rationale to enhance FLIPI with lymphopenia.

A minority of FL transforms into high-grade DLBCL, altering the indolent clinical course into that of an aggressive lymphoma with poor prognosis^11^. Multiple studies evaluated clinical risk factors including FLIPI and demonstrated an inconsistent ability to predict for transformation^12^. In our analysis, lymphopenia and FLIPI-L were independently associated with increased risk for transformation.

The precise mechanism of lymphopenia on FL prognosis and transformation is unclear and may be linked to immunogenicity. Immune evasion is one of the hallmarks of cancer and lymphomas are among the most immunogenic tumors. For instance, objective response rate (ORR) to programmed cell death 1 (PD-1) blockade ranges from 64% to 100% in Hodgkin’s lymphoma^9^. Immune checkpoint inhibitors were not extensively tested in FL, but a small cohort treated with nivolumab in a phase IB study demonstrated an ORR of 40%, highest among NHL. Lymphopenia may manifest in decreased tumor-specific T cell repertoire and thus compromised tumor immunity allowing immune escape by FL. Currently, use of PD-1 and programmed cell death ligand 1 (PD-L1) immunotherapy for FL remains limited; however, high content of PD-1-positive cells predicted for more favorable 5-year PFS/OS^9^ and was markedly decreased after histologic transformation to DLBCL^13^. This suggests a potential therapeutic window for PD-1/PD-L1 immunotherapy agents in patients at high risk for transformation.

The first-line management of FL ranges from observation, surgery, definitive radiotherapy, immunochemotherapy, to combined modality therapies. In our study, patients with the lowest FLIPI-L had excellent outcomes, with 5-year OS of 96%. In contrast, patients with the highest FLIPI-L demonstrate 5-year OS comparable to that of DLBCL patients receiving front-line therapy^14^. A prior study demonstrated a lower rate of complete response following rituximab, with worse time to progression in FL patients with lymphopenia^15^. Therefore, we conclude a role for treatment intensification in the high-risk FLIPI-L cohort by adding or increasing cycles of immunochemotherapy or newer immunotherapy agents for high FLIPI-L.

The ability for FLIPI-L to predict transformation can modify treatment approaches. The early recognition of a patient population who is at high risk for transformation could impact timing and initiation of more aggressive and life-prolonging therapy.

There are several key limitations in this study—its retrospective nature and long period of time over which patients were diagnosed and received treatment. Future prospective or multi-institutional validation will help its widespread implementation.

The FLIPI-L is a practical score utilizing a previously validated prognostic risk score, which adds lymphopenia, providing enhanced ability to predict OS, risk stratify, and prognosticate the risk of transformation.

## Supplementary information

Supplemental material table/figure legends

Supplemental figure 1

Supplemental table 1

Supplemental table 2

Supplemental table 3

Supplemental table 4
