# Supplementary material for: Enhancement of the Follicular Lymphoma International Prognostic Index (FLIPI) with lymphopenia (FLIPI-L): a predictor for overall survival and histologic transformation
Source: Blood Cancer J. 2020 Jan 2;9(12):104. doi: 10.1038/s41408-019-0269-6 (PMC6938796; doi:10.1038/s41408-019-0269-6)
Supplement: Supplementary file 1 — Supplemental material table/figure legends [file 41408_2019_269_MOESM1_ESM.docx]

**Supplemental Table 1**: a) FLIPI-L patient distribution b) FLIPI-L risk group

**Supplemental Table 2**: Cox-regression for OS: FLIPI and FLIPI-L

1. Cox regression for original FLIPI for overall survival
2. Cox regression for FLIPI-L score for overall survival

**Supplemental Table 3**: Binomial regression of FLIPI components predictive of transformation

**Supplemental Table 4**: FLIPI-L predictive of transformation

**Supplemental Figure 1**: AUC on 4-fold cross validation. Dark line represents AUC for FLIPI-ALS and grey line is original FLIPI
